# Establishment of a system for finding inhibitors of ε RNA binding with the HBV polymerase

**DOI:** 10.1111/gtc.12778

**Published:** 2020-06-08

**Authors:** Xiao‐Quan Liu, Eriko Ohsaki, Keiji Ueda

**Affiliations:** ^1^ Division of Virology Department of Microbiology and Immunology Osaka University Graduate School of Medicine Suita Japan

**Keywords:** epsilon RNA, HBV polymerase, packaging of pgRNA, terminal protein

## Abstract

Although several nucleo(s)tide analogs are available for treatment of HBV infection, long‐term treatment with these drugs can lead to the emergence of drug‐resistant viruses. Recent HIV‐1 studies suggest that combination therapies using nucleo(s)tide reverse transcriptase inhibitors (NRTIs) and non‐nucleo(s)tide reverse transcriptase inhibitors (NNRTIs) could drastically inhibit the viral genome replication of NRTI‐resistant viruses. In order to carry out such combinational therapy against HBV, several new NRTIs and NNRTIs should be developed. Here, we aimed to identify novel NNRTIs targeting the HBV polymerase terminal protein (TP)‐reverse transcriptase (RT) (TP‐RT) domain, which is a critical domain for HBV replication. We expressed and purified the HBV TP‐RT with high purity using an *Escherichia coli* expression system and established an in vitro ε RNA‐binding assay system. Then, we used TP‐RT in cell‐free assays to screen candidate inhibitors from a chemical compound library, and identified two compounds, 6‐hydroxy‐DL‐DOPA and N‐oleoyldopamine, which inhibited the binding of ε RNA with the HBV polymerase. Furthermore, these drugs reduced HBV DNA levels in cell‐based assays as well by inhibiting packaging of pregenome RNA into capsids. The novel screening system developed herein should open a new pathway the discovery of drugs targeting the HBV TP‐RT domain to treat HBV infection.

## Graphical abstract

HBVpol; TP‐spacer‐RT specifically binds with HBV packaging signal epsilon.

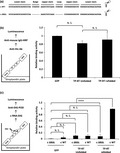

## 1 INTRODUCTION

Hepatitis B virus (HBV) is a main risk factor for the development of liver cirrhosis and hepatocellular carcinoma. The virus remains a major worldwide health problem, chronically infecting almost 350 million people (Schweitzer, Horn, Mikolajczyk, Krause, & Ott, 2015) and leading to approximately 686,000 deaths annually (Ward, Tang, Poonia, & Kottilil, 2016). Only limited therapies are currently available, such as peg‐interferon (peg‐IFN) and nucleo(s)tide reverse transcriptase inhibitors (NRTIs). However, interferons can cause severe side effects and NRTIs can lead to the emergence of NRTI‐resistant viruses (Tsukamoto et al., 2018).

Hepatitis B virus polymerase (HBVpol) consists of four domains—terminal protein (TP), spacer, reverse transcriptase (RT) and RNase H (RH) domains—and mediates diverse functions, including packaging of pregenome RNA (pgRNA) and protein priming for the initiation of reverse transcription, RNase H activity, and RNA‐ and DNA‐dependent DNA polymerization (Beck & Nassal, 2007; Sarin et al., 2016; Zoulim & Locarnini, 2009). The N‐terminal TP domain is conserved in all hepadnaviruses, but it is not found in the RT of any other species (Jones, Boregowda, Spratt, & Hu, 2012). The RT domain, including the YMDD motif, and RH catalytic domain of the polymerase are highly conserved in the RT of other species (Lanford, Kim, Lee, Notvall, & Beames, 1999). TP is a protein primer for HBV DNA replication, and tyrosine residue at Y63 of the TP has essential role for covalently linking with the first nucleotide in the viral minus‐strand DNA synthesis (Lanford, Notvall, Lee, & Beames, 1997; Weber et al., 1994; Zoulim & Seeger, 1994); and both the TP and RT domains have been reported to be required for binding and packaging of pgRNA into nucleocapsids through the interaction of TP‐RT with epsilon (ε) RNA (Jones, Clark, Cao, Tavis, & Hu, 2014; Stahl, Beck, & Nassal, 2007), a packaging signal of HBV pgRNA.

The ε RNA sequences are composed of approximately 60 nucleotides and are located at both the 5' and 3' ends of pgRNA; the 5' ε of the pgRNA is important for pgRNA packaging and initiation of minus‐strand DNA synthesis (Hu & Boyer, 2006; Rieger & Nassal, 1996; Wang, Wen, & Nassal, 2012). It has highly conserved structural features, that is, a lower stem, a central bulge and an upper stem with the apical loop (Hu & Boyer, 2006). The central bulge and the apical loop structures of the ε RNA (Lanford et al., 1997; Zoulim & Seeger, 1994) sequences are important for the functional protein priming (Jones et al., 2012). During reverse transcription, the polymerase is recruited to the upper stem located at the 5' end of ε, and then the TP domain of polymerase binds with the conserved bulged domain and initiates protein priming of the viral minus‐strand DNA synthesis (Hu & Seeger, 2015; Jones et al., 2012). This is a crucial step for HBV replication, and therefore, it should be very attractive for use in the development of a system to identify compounds that inhibit ε RNA‐polymerase interaction.

A recent study reported that carbonyl J acid derivatives blocked the duck hepatitis B virus (DHBV) protein priming by inhibiting the DHBV polymerase interaction with ε RNA (Wang et al., 2012). It could open a new avenue, if we developed a high‐throughput screening (HTS) system for ε RNA‐polymerase binding inhibitors. For this purpose, high expression and purification of polymerase should be an essential step. However, although many research groups have tried to purify the full length of HBV polymerase protein, it has proven difficult to achieve the highly purified, full‐length protein in high yield.

Here, we succeeded in expressing and purifying the TP‐spacer‐RT region (TP‐RT) with high purity using an *Escherichia coli* expression system and developed an in vitro cell‐free assay system to detect the specific interaction between TP‐RT and ε RNA. Using this assay system, we found two candidate inhibitors, the 6‐hydroxy‐DL‐DOPA (DL‐DOPA) and N‐oleoyldopamine (OLDA), that blocked the binding TP‐RT with ε RNA from LOPAC^®1280^ (Sigma‐Aldrich LO4200). These drugs also showed to reduce HBV DNA amplification by inhibiting pgRNA packaging into capsids in cell‐based HBV infection and/or amplification systems and could serve as a new type of anti‐HBV agents, though surface plasmon resonance study showed that DL‐DOPA binding to TP‐RT seemed to be nonspecific. It is necessary for sure to remove cccDNA in order to eliminate HBV completely (Chisari, Mason, & Seeger, 2014; Hui et al., 2006; Lucifora & Protzer, 2016). The system developed herein, however, will contribute to the identification of new anti‐HBV drugs that specifically inhibit the packaging of pgRNA into capsids by blocking the interaction between ε RNA and HBV polymerase.

## 2 RESULTS

### 2.1 Expression and purification of a recombinant TP‐spacer‐RT_1‐690_ protein in *E. coli*

**Figure 1:**
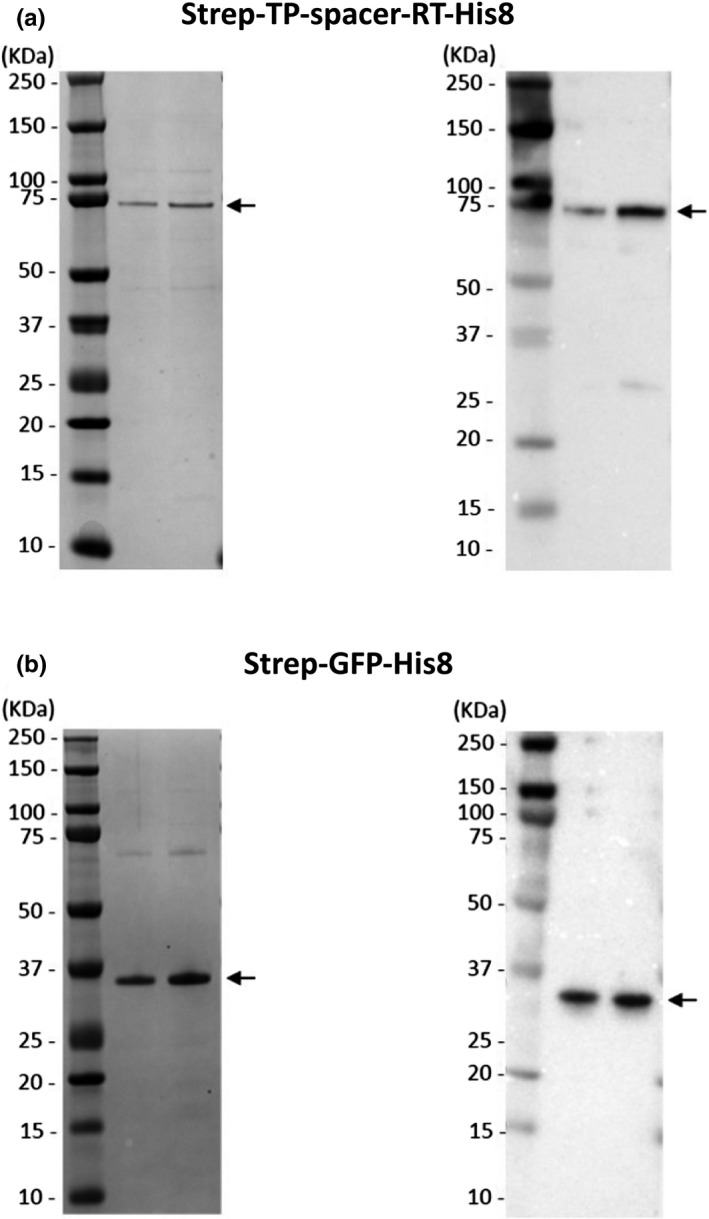
Purification of a recombinant Strep‐TP‐spacer‐RT‐His8 and a Strep‐GFP‐His8 control protein. (a) Left panels: CBB stained the Strep‐TP‐spacer‐RT‐His8 (100 ng [central] and 200 ng [right]), and (b) Strep‐GFP‐His8 proteins (250 ng [central] and 500 ng [right]) in an SDS‐PAGE. 1The final products of the Strep‐TP‐spacer‐RT‐His8 and the Strep‐GFP‐His8 protein showed over 90% purity. Right panels: Western blots for the Strep‐TP‐spacer‐RT‐His8 and Strep‐GFP‐His8 proteins shown in the corresponding left panels. For Western blotting, an anti‐His Ab against the His tag was used to detect

Strep‐TP‐spacer‐RT‐His8 (HBVpol 1‐690aa) and Strep‐GFP‐His8 were expressed in the Rosetta‐gami2 (DE3) *E. coli* strain and purified using a nickel column as described in the Experimental Procedures. We chose a denaturing condition, since purification of this protein under a nondenaturing condition gave a very low yield. Protein purity was examined by CBB staining (see the left panels in Figure 1a,b) and confirmed by Western blotting with an anti‐His‐tag antibody (right panels in Figure 1a,b). As shown in the figure, Strep‐TP‐spacer‐RT‐His8 and Strep‐GFP‐His8 proteins of more than 90% purity were obtained, respectively.

### 2.2 The purified Strep‐TP‐spacer‐RT‐His8 protein shows a specific ε‐binding activity

**Figure 2:**
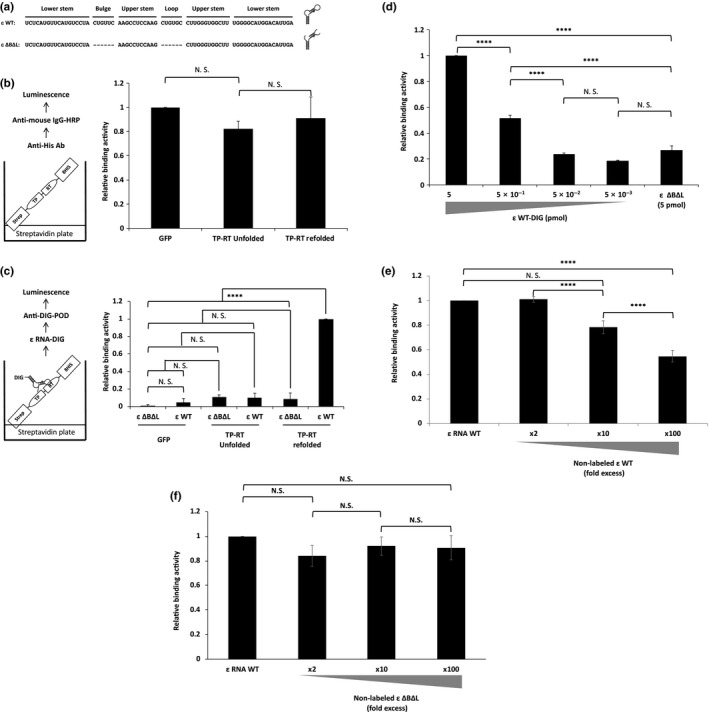
ε RNA‐binding assay with the purified Strep‐TP‐spacer‐RT‐His8 and Strep‐GFP‐His8. (a) An ε RNA wild‐type (ε WT) and the mutant (ε ∆B∆L) RNA sequence used in this experiment. (b) Left panel: the principle of the binding evaluation of the Strep‐TP‐spacer‐RT‐His8 (TP‐RT) and the Strep‐GFP‐His8 (un‐refolded or refolded) proteins on a streptavidin plate. The bound protein was evaluated with a mouse monoclonal anti‐His tag antibody followed by an anti‐mouse IgG conjugated with HRP. The HRP was measured as luminescence. Right panel: the relative binding activity of the anti‐mouse IgG conjugated with HRP to Strep‐GFP‐His8 and Strep‐TP‐spacer‐RT‐His8 (un‐refolded or refolded) proteins bound on a streptavidin plate. The relative binding activity was determined as follows: relative binding activity = (X‐NC)/(GFP‐NC), where X = each sample, NC = negative control (without antibodies). (c) Left panel: the principle of the ε RNA‐binding assay with Strep‐GFP‐His8 and Strep‐TP‐spacer‐RT‐His8 (TP‐RT) (un‐refolded or refolded) proteins. 5 pmol of DIG‐labeled either ε WT or ε ∆B∆L was added to the reaction. The DIG‐labeled bound RNA was detected with an anti‐DIG antibody conjugated with HRP. The HRP was measured as luminescence. Right panel: the relative binding activity of the respective Strep‐GFP‐His8 and Strep‐TP‐spacer‐RT‐His8 (TP‐RT) (un‐refolded or refolded) protein with a wild‐type ε RNA (ε WT) and the mutant ε RNA (ε ΔBΔL). The relative binding activity in this experiment was determined as follows: relative binding activity = (X‐NC)/ (TP‐RT refolded epsilon WT‐N/C), where X = each sample, NC = negative control (without epsilon RNA). (d) An ε RNA‐dependency assay. Initial 5 pmol of DIG‐labeled ε WT was serially diluted every 10‐fold, and the binding was measured. Binding with ε ΔBΔL (5 pmol) was also tested. (e and f) A competition assay of ε WT binding with the purified strep‐TP‐spacer‐RT‐His8 protein. Competitor of nonlabeled either ε WT (e) or ε ΔBΔL (f) was added to the reaction at the ×2, ×10, ×100 excess (10, 50 and 500 pmol, respectively), and the bound DIG‐labeled ε WT was measured as luminescence as described above. Data were obtained from at least three independent experiments, and the mean value and the S.D. are shown as relative values to the control (*p < .05, **p < .01, ***p < .005, ****p < .001, N.S.: not significant). The relative binding activity in (d), (e) and (f) was determined as follows: relative binding activity = (X‐NC)/ (5 pmol ε WT‐NC), where X = each sample. NC = negative control (without ε RNA)

We next examined whether the purified Strep‐TP‐spacer‐RT‐His8 exhibited in vitro ε RNA‐binding activity. The refolded Strep‐TP‐spacer‐RT‐His8 and Strep‐GFP‐His8 proteins were fixed on a streptavidin‐coated plate as described in the Experimental Procedures. The protein bound the plate was confirmed with an anti‐His‐tag antibody followed by an anti‐mouse IgG conjugated with HRP for detection by luminol reaction (Figure 2b). As shown in Figure 2c, the ε WT (Figure 2a upper) but not the mutant ε RNA, ε ∆B∆L (Figure 2a lower) bound with the Strep‐TP‐spacer‐RT‐His8 protein. The binding activity was clearly high for a refolded protein (Figure 2c). This result was consistent with previous studies which reported that the central bulge of ε RNA was essential for the polymerase binding (Jones et al., 2012). We initially constructed and purified TP alone as His‐SUMO‐TP and its control His‐SUMO (Figure S1a,b), and performed a similar assay. TP alone, however, did not show any specific ε WT‐binding activity as previously reported (Hu & Boyer, 2006) (Figure S1c,d).

To further confirm the specific binding activity, we performed an ε RNA‐dependency assay and a competition assay. In case of the ε RNA‐dependency assay, the amount of ε WT was serially diluted by a factor of 10. The bound ε WT clearly decreased stoichiometrically (Figure 2d).

In the competition analysis, addition of the ε WT as a competitor reduced the binding activity of the DIG‐labeled ε WT in a dose‐dependent manner (Figure 2e) but not the mutant ε ∆B∆L (Figure 2f). These results strongly revealed that the Strep‐TP‐spacer‐RT protein should have an in vitro ε RNA‐binding activity and an in vitro ε RNA‐binding assay system should be assembled; such a system would be a rapid and reliable means of identifying anti‐HBV agents as inhibitors against ε RNA‐binding activity. Therefore, we applied this assay for chemical compounds screening.

Although replication activity with poly(dA)/oligo(dT_7_) or poly(rA)/oligo(dT_7_) with substrates was also tested with the purified Strep‐TP‐spacer‐RT‐His8, we could not detect any polymerizing activity (data not shown).

### 2.3 Screening of small‐molecule compounds to find inhibitors of Strep‐TP‐spacer‐RT‐His8 binding with ε RNA

**Figure 3:**
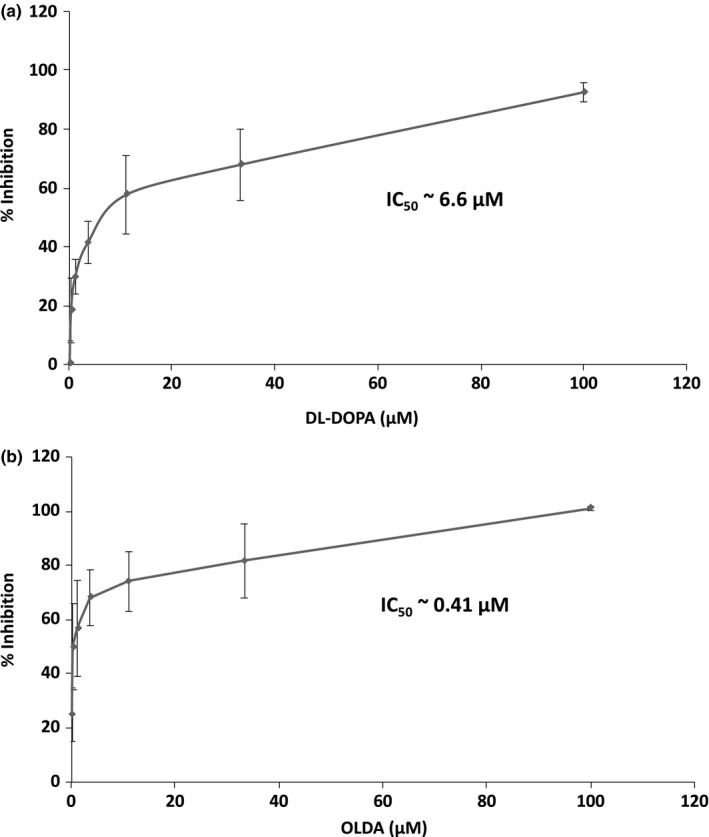
Inhibition of ε RNA binding with the purified Strep‐GFP‐His8 and Strep‐TP‐spacer‐RT‐His8 proteins by 6‐hydroxy‐DL‐DOPA (DL‐DOPA) and N‐oleoyldopamine (OLDA). Among the 1,280 chemical compounds, DL‐DOPA and OLDA were picked up as candidate inhibitors of ε RNA binding with the Strep‐TP‐spacer‐RT‐His8 protein. Each drug was added at the concentration shown in the panel (a and b, respectively). Based on the bound ε RNA, the inhibitory activity was calculated and shown as % inhibition. The % inhibition was calculated as follows: %inhibition = 100–(X–NC)/ (ε WT–NC) × 100, where X = each drug, NC = negative control (without ε RNA). The IC50 was calculated as follows: IC50 = 10^E[Log(A/B) × (50–C)/(D–C) + Log(B)] (A: the first concentration of the compound exceeding 50% inhibition; B: the first concentration of the compound less than 50% inhibition; C: the inhibition percentage of B; D: the inhibition percentage of A). Data were obtained from at least three independent experiments, and the mean value and the S.D. are shown

Next, we applied this assay to screen chemical compounds to identify novel inhibitors of Strep‐TP‐spacer‐RT‐His8 binding with ε RNA. A small‐molecule compound library (LOPAC^®1280^; Sigma‐Aldrich LO4200) was used for this experiment. Primary screening was performed at 100 µM of each compound, and then candidate inhibitors that showed over 80% inhibition were retested and their 50% inhibition concentration (IC_50_) values were determined. Eight compounds showed more than 80% inhibition. To check their inhibition specificities, all eight compounds were made serial 3‐time dilution for dose‐dependent experiment and found that only two compounds exhibited dose‐dependent inhibition of the binding (data not shown). Therefore, in the final analysis, 2 of the 1,280 compounds, 6‐hydroxy‐DL‐DOPA (DL‐DOPA) and N‐oleoyldopamine (OLDA), were picked up as candidate inhibitors of ε RNA binding with Strep‐TP‐spacer‐RT‐His8 (Figure S2a). The putative IC_50_ values of DL‐DOPA and OLDA were around 6.6 and 0.41 µM, respectively, by calculation. The % inhibition seemed to be linear to log scale of the drug dose (Figure 3a,b).

### 2.4 6‐hydroxy‐DL‐DOPA and N‐oleoyldopamine inhibit HBV DNA amplification in the cell‐based assay

**Figure 4:**
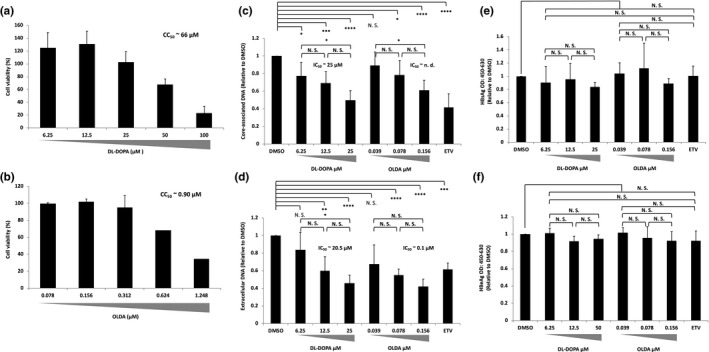
6‐hydroxy‐DL‐DOPA (DL‐DOPA) and N‐oleoyldopamine (OLDA) inhibit HBV amplification in a cell line stably producing HBV. The HB611 cells were treated with either DL‐DOPA or OLDA or 20 nM entecavir (ETV). (a and b) The HB611 cell viability was checked in the presence of either OLDA or DL‐DOPA with different concentrations as shown. CC_50_ = 10ˆE (E = log [A/B] × [50–C]/[D–C] +log [B], where A = the first drug concentration exceeding 50% cytotoxicity, B = the first drug concentration to show just lower than 50% cytotoxicity. C = %Cytotoxicity‐lower [%Cytotoxicity of B], and D = % Cytotoxicity ‐higher [%Cytotoxicity of C]). (c and d) Nine days after treatment of each drug at different concentrations, core‐associated and extracellular particle‐associated HBV DNA were extracted and quantified by qPCR. (e and f) Nine days after treatment of each drug at different concentrations as shown, HBeAg and HBsAg in the soup were measured with each ELISA kit, respectively. The IC_50_ was calculated as described in the Figure 3 legend. Data were obtained from at least three independent experiments, and the mean values and the S.D. are shown as relative values to the control (*p < .05, **p < .01, ***p < .005, ****p < .001, N.S.: not significant)

Our in vitro cell‐free ε RNA‐binding assay system nominated two candidates to block ε RNA binding with Strep‐TP‐spacer‐RT‐His8 protein. Then, we evaluated the inhibition effects on HBV DNA amplification in a cell‐based HBV amplification or an infection system (Figure 4). HB611 cells, in which three tandemly arranged HBV genomes were integrated into a cellular chromosome and persistently produced HBV (Tsurimoto, Fujiyama, & Matsubara, 1987), and human NTCP expressing HepG2 (NTCP/G2) cells as an HBV infection system were utilized (Iwamoto et al., 2014).

First of all, cytotoxicity of the compounds was tested to determine the dose for the experiment and the 50% cytotoxicity concentration (CC_50_) (Figure 4a). As a result, putative CC_50_ values of DL‐DOPA and OLDA were about 66 and 0.90 µM, respectively, in case of HB611 cells (Figure 4a,b). We used the maximum compound concentration which showed over 90% cell viability. Within the range of drug concentration, NTCP/G2 and HB611 cells appeared not to be damaged (Figure S3).

Intracellular core‐associated and extracellular particle‐associated DNA were prepared for quantification analysis by qPCR. As shown in Figure 4c,d, both core‐associated and extracellular particle‐associated HBV DNA in HB611 cells were decreased by DL‐DOPA and OLDA treatment in a dose‐dependent manner. The putative IC_50_ values of DL‐DOPA for core‐associated DNA and extracellular DNA were around 25 and 21 µM, and those of OLDA could be more than 0.16 and 0.10 µM, respectively. On the other hand, HBsAg and HBeAg production were not changed by these compounds (Figure 4e,f). These results suggested that DL‐DOPA and OLDA should specifically inhibit HBV DNA amplification process such as packaging and genome DNA synthesis but not transcription.

**Figure 5:**
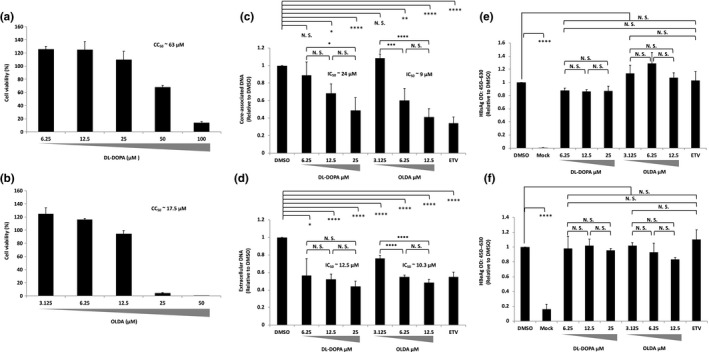
6‐hydroxy‐DL‐DOPA (DL‐DOPA) and N‐oleoyldopamine (OLDA) inhibit HBV amplification in an HBV infection system. (a and b) The NTCP/G2 cell viability was tested at the different concentrations of OLDA or DL‐DOPA, respectively. (c and d) NTCP/G2 cells were infected with 500 GEI of HBV in the presence of each drug as indicated. 20 nM entecavir (ETV) was also tested as an HBV replication inhibitor. Core‐associated DNA and extracellular particle‐associated HBV DNA were extracted at the end and quantified by qPCR. (e and f) HBeAg and HBsAg in the soup were checked by respective ELISA kit. Mock: noninfected negative control. IC_50_ and CC_50_ were determined as described in the Figure 3 and the Figure 4 legend, respectively. Data were obtained from at least three independent experiments, and the mean values and the *SD* are shown as relative values to the control (*p < .05, **p < .01, ***p < .005, ****p < .001, N.S., not significant)

Effect of the nominated compounds was also tested in an HBV infection system using HepG2 cells expressing human NTCP (NTCP/G2) (Iwamoto et al., 2014). Putative CC_50_ values of DL‐DOPA and OLDA were about 63 and 18 µM, respectively (Figure 5a,b). As for DL‐DOPA, the CC_50_ value of NTCP/G2 was consistent with the value in HB611, but that of OLDA was approximately 20‐fold higher than that in HB611. As similar with the HB611 cells, we used the maximum concentration which showed over 90% viability in the infection assay. As shown in Figure 5c,d, DL‐DOPA and OLDA decreased both core‐associated and extracellular particle‐associated HBV DNA levels in a dose‐dependent manner. The putative IC_50_ values of DL‐DOPA for core‐associated DNA and extracellular DNA were around 24 and 13 µM and those of OLDA were around 9.0 and 10 µM, respectively, though more wide range of drug concentration was required for the accurate determination. Since HBsAg and HBeAg production were not changed by DL‐DOPA and OLDA (Figure 5e,f), it was suggested that two compounds should have an inhibition effect not on HBV entry process including attachment to cccDNA formation but on packaging and/or DNA replication in the HBV life cycle.

### 2.5 6‐hydroxy‐DL‐DOPA and N‐oleoyldopamine inhibit HBV pgRNA packaging

**Figure 6:**
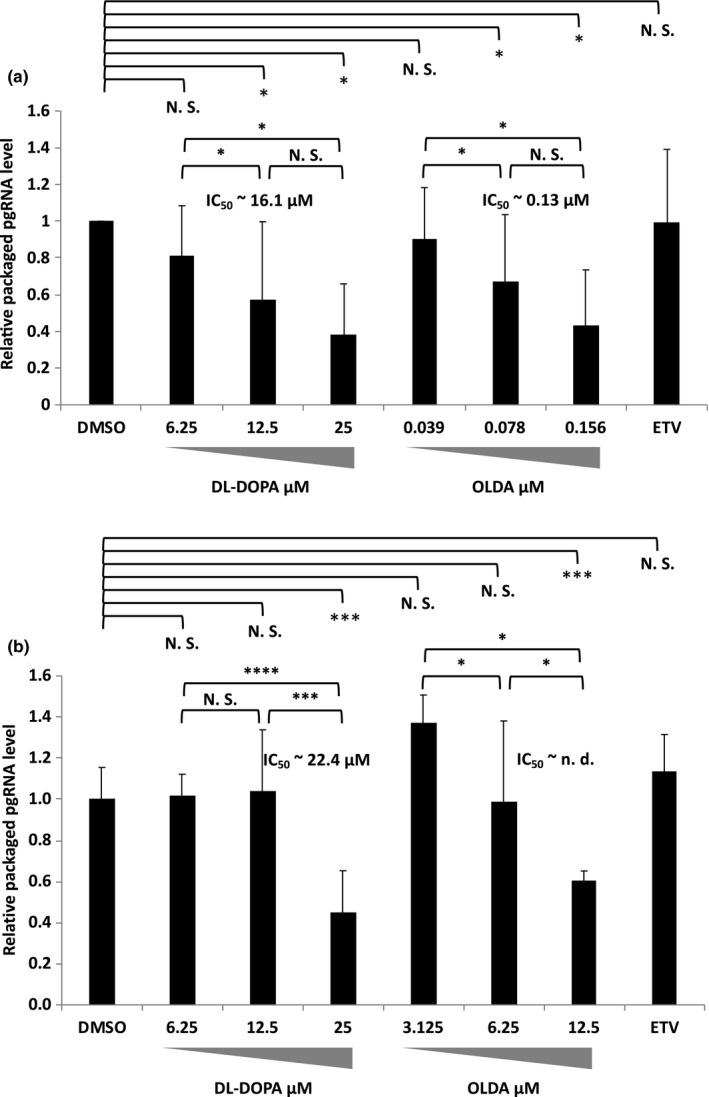
Quantification of packaged HBV pgRNA. (a) HB611 cells were treated with either 6‐hydroxy‐DL‐DOPA (DL‐DOPA) or N‐oleoyldopamine (OLDA) or 20 nM entecavir (ETV) as indicated. At the last day of the assays, core‐associated RNA was extracted and reversely transcribed as described in the Experimental Procedures. Then, cDNA was quantified by qPCR and the relative packaging efficiency to the control (DMSO) was calculated as each RT‐qPCR value/ that of the control (DMSO) RT‐qPCR. NTCP/G2 cells were infected with 500 GEI of HBV in the presence of either N‐oleoyldopamine or 6‐hydroxy‐D L‐DOPA as indicated. 20 nM entecavir (ETV) was also tested as an HBV replication inhibitor but not a packaging inhibitor. (b) NTCP/G2 cells were infected with 500 GEI of HBV in the presence of either 6‐hydroxy‐DL‐DOPA (DL‐DOPA) or N‐oleoyldopamine (OLDA) as indicated. 20 nM entecavir (ETV) was also tested as an HBV replication inhibitor but not a packaging inhibitor. IC_50_ was determined as described in the Figure 3 legend. Data were obtained from at least three independent experiments, and the mean values and the S.D. are shown as relative values to the control (*p < .05, **p < .01, ***p < .005, ****p < .001, N.S., not significant)

DL‐DOPA and OLDA were nominated as inhibitors of ε RNA binding with TP‐RT. Thus, we tested whether pgRNA packaging into core particles was really inhibited by these drugs. Core‐associated RNA was extracted with an RNA extraction reagent, and reverse transcription followed by qPCR (RT‐qPCR) was performed. As shown in Figure 6a (NTCP/G2 infection system) and 6B (HB611), both drugs were supposed to be inhibitors of packaging as expected without significant change of sAg and/or eAg expression (Figures 4 and 5e,f). OLDA seemed to be the more effective as packaging inhibitors, though the dose‐dependency was clearer for OLDA. As for entecavir, it is a nucleotide analog to inhibit HBV DNA synthesis. Therefore, it would be expected that the amount of relative packaged RNA should be increased by entecavir. In our case, such tendency was observed slightly but not significant, probably because these HBV infection and amplification system were not so efficient for HBV amplification. Or otherwise, if the higher dose of entecavir were used, the clearer increase might be observed.

### 2.6 Binding between DL‐DOPA and Strep‐TP‐spacer‐RT‐His8 is rather nonspecific

We tried to determine affinity between the Strep‐TP‐spacer‐RT‐His8 and the nominated compounds, DL‐DOPA and OLDA, by surface plasmon resonance (SPR). As a result, DL‐DOPA and OLDA showed relatively high affinity with TP‐RT, whose *K*D values were 11 and 72 nM, respectively (Figure S4a,b). Entecavir and dopamine never showed affinity with the Strep‐TP‐spacer‐RT‐His8 as expected (Figure S4c,d). On the other hand, DL‐DOPA unexpectedly showed high affinity with Strep‐GFP‐His8 (Figure S4e), but not OLDA (Figure S4f).

## 3 DISCUSSION

Hepatitis B virus polymerase has many important functions in the HBV replication cycle, including viral RNA binding (Beck & Nassal, 2003; Wang, Qian, Guo, & Hu, 2003), encapsidation (Bartenschlager & Schaller, 1992; Hirsch, Lavine, Chang, Varmus, & Ganem, 1990; Hirsch, Loeb, Pollack, & Ganem, 1991; Junkerniepmann, Bartenschlager, & Schaller, 1990; Knaus & Nassal, 1993; Pollack & Ganem, 1993), protein priming (Jones et al., 2012; Lanford et al., 1999), template switching (Liu, Tian, & Loeb, 2003), reverse transcription (Summers & Mason, 1982), RNA degradation (Clark & Hu, 2015) and plus‐strand DNA synthesis (Hu & Boyer, 2006; Jones et al., 2012; Jones et al., 2014). ε RNA, a packaging signal for HBV encapsidation of pgRNA, forms a stem‐loop secondary structure, is recognized by the HBV polymerase and is used as the initial template for the reverse transcription (Hu, Flores, Toft, Wang, & Nguyen, 2004; Hu, Toft, Anselmo, & Wang, 2002; Hu, Toft, & Seeger, 1997; Jones et al., 2012).

Although a completely effective HBV treatment would require the elimination of cccDNA, we aimed to develop an in vitro binding assay system by using a partial domain of polymerase, TP‐spacer‐RT, to detect a specific binding activity with ε RNA and find novel compounds inhibiting the activity, because it was reported that TP‐spacer‐RT domain was a critical and a sufficient domain for polymerase ε RNA binding and protein priming (Jones et al., 2014), and finding inhibitors of ε and HBVpol TP‐spacer‐RT binding could give us novel options for treatment of HBV infection. For this purpose, we purified the TP‐spacer‐RT domain with a streptactin tag at the N‐terminus and a histidine octamer (His8) at the carboxy terminus (Strep‐TP‐spacer‐RT‐His8) using an *E. coli* expression system, and obtained the protein with over 90% purity at the CBB‐staining level (Figure 1). To evaluate the specificity and the reliability of this protein, we performed a binding assay using wild‐type and mutant ε RNA, and control proteins (Figure 2a‐c), and demonstrated specific ε RNA binding with Strep‐TP‐spacer‐RT‐His8 in a dose‐dependent manner (Figure 2d,e). Thus, it was suggested that this assay system should detect specific Strep‐TP‐spacer‐RT‐His8 binding with ε RNA. On the other hand, it was reported that the RT domain alone bound with RNA without sequence specificity (Nassal & Rieger, 1996). In contrast, our purified Strep‐TP‐spacer‐RT‐His8 protein was capable of recognizing only the wild‐type ε RNA, but not the central bulge‐ and apical loop‐deleted mutant (ε ΔBΔL), suggesting that this assay system should reflect the previous results (Jones et al., 2012) and be useful to detect specific TP‐spacer‐RT and ε RNA binding.

Screening for inhibitors which prevented the binding of Strep‐TP‐spacer‐RT‐His8 with ε RNA identified two compounds, DL‐DOPA and OLDA, from the 1,280 small‐molecule compounds. These two compounds represented a specific inhibitory effect in the cell‐free assay and on HBV replication and/or packaging in a stable HBV production system (HB611) and in an HBV infection system using NTCP/G2. Putative specific index (SI) values (CC_50_/IC_50_) of DL‐DOPA in HB611 cells were about 2.6 (core‐associated DNA) and about 3.1 (particle‐associated DNA) and those of OLDA were <5.6 (core‐associated DNA) and about 9.0 (particle‐associated DNA) (Table 1). In the infection system, putative SI values of DL‐DOPA were about 2.6 (core‐associated DNA) and about 4.8 (particle‐associated DNA) and again those of OLDA were about 2.0 (core‐associated DNA) and about 1.8 (particle‐associated DNA) (Table 1). It should be probably because drug toxicity and/or uptake, and virus trafficking from intracellular to extracellular could be different dependent on cells so that IC50 of the compounds would differ dependent on the cell type and in case of DL‐DOPA, it seemed that the binding activity with Strep‐TP‐spacer‐RT‐His8 should be nonspecific which reflected rather a weak dose‐dependent inhibition of pgRNA packaging (Figure 6 and Figure S4).

**Table 1:** Putative CC_50_, IC_50_ and SI values

		CC_50_ (µM)	IC_50_ (µM)		Selectivity index (SI) values	
			Core‐associated DNA	Particle‐associated DNA	Core‐associated DNA	Particle‐associated DNA
HB611 cells	DL‐DOPA	66	25	21	2.6	3.1
	OLDA	0.90	*n*.d.	0.10	<5.6	9.0
NTCP/G2 cells	DL‐DOPA	63	24	13	2.6	4.8
	OLDA	18	9	10	2.0	1.8

Production of HBeAg and HBsAg was not affected by these compounds (Figures 4 and 5) and in contrast, packaging of pgRNA into core particles was inhibited by the drugs, suggesting that 2 compounds should inhibit the HBV genome replication by inhibiting binding of TP‐spacer‐RT with ε RNA followed by packaging and reverse transcription, though the drugs should be improved to reduce the cytotoxicity.

Surprisingly, our screening assay identified two compounds containing dopamine‐like structure (Figure S2b), although there was low similarity between these two compounds except a shared tyrosine bone structure. DL‐DOPA was found to be an inhibitor of apurinic/apyrimidinic endonuclease (APE1) repair activity via the inhibition of its AP site cleavage activity (Simeonov et al., 2009). The report showed that APE1 played an essential role in protecting cells from accumulation of endogenously arising basic DNA lesions (Fung & Demple, 2005). Another report suggested that DL‐DOPA appeared to disrupt the oligomeric ring structure of RAD52, which is important for DNA double‐strand break repair and homologous recombination, and prevented its recruitment to single‐strand DNA. Both of reports suggested that DL‐DOPA had inhibitory effects on DNA damage repair (Chandramouly et al., 2015). One possibility would be that a DNA repair system of this type participated in the HBV DNA replication, and DL‐DOPA might inhibit HBV DNA replication by inhibiting its repair machineries.

N‐oleoyldopamine is a bioactive amide of a fatty acid originally found in the mammalian brain and is considered to be an endogenous capsaicin‐like lipid that acts as an agonist of the capsaicin receptor, VR1 (Chu et al., 2003; Spicarova & Palecek, 2010). Interestingly, our recent study identified capsaicin as a novel retroviral RT inhibitor (Nishikawa et al., 2018). OLDA has a benzene ring similar to that of capsaicin, although the partial side group is different. It is interesting to note that DL‐DOPA also has a similar benzene ring (Figure S2b). Therefore, both DL‐DOPA and OLDA might affect the RT function due to their structural similarity with capsaicin.

Affinity analysis of Strep‐TP‐spacer‐RT‐HIS8 and Strep‐GFP‐HIS8 with compounds by SPR, however, suggested that DL‐DOPA and OLDA should have their own characters, that is, unexpectedly DL‐DOPA bound with Strep‐GFP‐HIS8 as well as Strep‐TP‐RT‐HIS8 at relatively high affinity, *K*D of which was 11 and 8.4 nM, and the compound showed some inhibitory effect on pgRNA packaging followed by HBV genome replication in the cell‐based assay. The nonspecific binding should reflect that the compound needed rather higher concentration to inhibit HBV replication in the cell‐based assay compared to those in SPR analysis. Therefore, OLDA should more specifically inhibit pgRNA packaging into the capsids followed by reverse transcription and (+)‐strand DNA synthesis to complete HBV genome replication.

Taken together, we successfully purified HBV polymerase TP‐spacer‐RT domain expressed in *E. coli* with high purity and established a system to find new compounds or chemicals to inhibit binding of TP‐spacer‐RT with a packaging signal of ε RNA. We found two compounds, DL‐DOPA and OLDA, to inhibit pgRNA packaging into the capsids followed by HBV genome replication with the assay system. Although these chemicals would not be applicated for clinical use directly, our system should be very useful to explore new anti‐HBV agents in terms of packaging inhibitors.

## 4 EXPERIMENTAL PROCEDURES

### 4.1 Protein expression and purification

The TP‐spacer‐RT domain (amino acids 1 to 690) of HBVpol was inserted into the pQE‐Tri System His‐Strep 2 vector (Qiagen) to construct pQE‐Tri‐TP‐spacer‐RT. In this vector, the N‐terminus was streptactin (strep)‐tagged and the C‐terminus was histidine octamer (His8)‐tagged, and thus, the Strep‐TP‐spacer‐RT‐His8 protein was designed to express. An *E. coli* strain, Rosetta‐gami B (DE3) pLysS (Pierce), was transformed with the pQE‐Tri‐TP‐spacer‐RT, and the cells were grown at 37°C to an OD_600_ of 0.3 and were induced for protein expression with 0.5 mM IPTG at 30°C for 12 hr. The *E. coli* pellets were suspended in a lysis buffer (100 mM Tris‐HCl [pH 8.0], 0.1% Triton X‐100^®^, 300 mM NaCl, 0.1% protease inhibitor cocktail [Sigma‐Aldrich] and 1% SDS) and sonicated for 20 s five times with 30‐s intervals on ice. The cell lysate was left to stand at 4°C overnight and then centrifuged at 12,000× *g* for 40 min to remove the SDS. The supernatant was passed through with a 0.45‐µm filter (Millipore) and incubated with Profinity IMAC^®^ Ni‐charged resin (Bio‐Rad) to purify the Strep‐TP‐spacer‐RT‐His8 by affinity to the resin. The protein‐bound resin was washed with a washing buffer (100 mM Tris‐HCl [pH 8.0], 0.1% sarkosyl, 300 mM NaCl, 0.1% protease inhibitor cocktail [Sigma‐Aldrich] and 5 mM imidazole) five times and then eluted with an elution buffer (100 mM Tris‐HCl [pH 8.0], 0.1% sarkosyl, 300 mM NaCl, 0.1% protease inhibitor cocktail and 150 mM imidazole). The purified proteins were supplemented with 2 mg/ml NV10^®^ (Expedeon) and incubated for overnight at room temperature (R.T.) for refolding in a buffer containing (100 mM Tris‐HCl [pH 8.0], 300 mM NaCl, 0.1% protease inhibitor cocktail and 1 mM dithiothreitol [DTT]) by using Slide‐A‐Lyzer dialysis cassette system (10 kDa) (Pierce).

A pQE‐Tri‐GFP plasmid was also constructed to express a control protein. This plasmid was designed to express Strep‐GFP‐His8. The protein was expressed in the same way. The cell pellet was suspended in another lysis buffer (50 mM NaH_2_PO_4_ [pH 8.0], 0.1% Triton X‐100^®^, 300 mM NaCl, 1 mM 2‐mercaptoethanol, 1 mg/ml lysozyme, 125U of benzonase^®^ [Novagen] and 0.1% protease inhibitor [Sigma‐Aldrich]) and sonicated for 20 s five times with 30‐s interval on ice. The cell lysate was centrifuged at 12,000× *g* for 40 min at 4°C. The supernatant was passed through a 0.45‐µm filter (Millipore), and Strep‐GFP‐His8 was then purified using the Profinity IMAC^®^ Ni‐charged resin as Strep‐TP‐spacer‐RT‐His8. The resin bound with Strep‐GFP‐His8 was washed with a washing buffer (50 mM NaH_2_PO_4_ [pH 8.0], 0.1% Triton X‐100^®^, 300 mM NaCl, 10% glycerol, 5 mM imidazole, 0.1% protease inhibitor) five times, and then the bound Strep‐GFP‐His8 was eluted with an elution buffer (50 mM NaH_2_PO_4_ [pH 8.0], 0.1% Triton X‐100^®^, 300 mM NaCl, 10% glycerol, 150 mM imidazole and 0.1% protease inhibitor) five times.

### 4.2 Preparation of epsilon (ε) RNA

The wild‐type and mutant ε RNAs (Hu & Boyer, 2006) were synthesized by a manufacturer. Both RNAs labeled with DIG at the 3'‐end and nonlabeled RNAs were synthesized. The sequence of the ε RNAs was as follows: wild‐type (ε WT), 5'‐UCUCAUGUUCAUGUCCUACUGUUCAAGCCUCCAAGCUGUGCCUUGGGUGGCUUUGGGGCAUGGACAUUGA − 3'; the central bulge‐ and the apical loop‐deleted mutant (ε ΔBΔL), 5'‐UCUCAUGUUCAUGUCCUAGCCUCCAAUUGGGUGGCUUUGGGGCAUGGACAUUGAC‐3'. Each RNA (100 µM) was self‐annealed according to the following protocol: 95°C for 5 min, 90°C for 3 min, 80°C for 3 min, 75°C for 3 min, 70°C for 3 min, 65°C for 3 min, 60°C for 3 min, 55°C for 3 min, 50°C for 3 min, 45°C for 3 min, 40°C for 3 min, 35°C for 3 min, 30°C for 3 min, and 25°C for 5 min and kept at 4°C. After this process, the RNA was stored at −70°C until use.

### 4.3 In vitro ε RNA‐binding assay

For the ε RNA‐binding assay using Strep‐ and His8‐tagged proteins, 50 pmol of the purified proteins were fixed on a streptavidin‐coated 96‐well plate (Pierce) and were incubated at R.T. overnight. Then, 100 µl of 1 mg/ml BSA in phosphate‐buffered saline (PBS) and 50 pmol/well of desthiobiotin (Sigma‐Aldrich) were added and incubated for 1 hr at 37°C to block nonspecific binding and mask unbound streptavidin. Next, 5 pmol DIG‐labeled RNA of ε WT or ε ΔΒΔL was added to the protein‐coated plate in a 100 µl reaction buffer (50 mM Tris‐HCl [pH 7.5], 150 mM NaCl, 1 mM EDTA, 0.05% NP 40^®^, 1 mg/ml BSA, 1 µg/ml poly(I/C), 0.1% protease inhibitor cocktail [Sigma‐Aldrich] and 2 mM DTT) based on the previous assay condition with some modification (Clark, Jones, & Hu, 2017; Jones et al., 2012). After 3 hr of incubation at R.T., each well was washed with a 250 µl wash buffer (100 mM Tris‐HCl [pH 8.0], 300 mM NaCl, 1 mM MgCl_2_, 5 mM 2‐mercaptoethanol and 50 µg/ml BSA) three times, and then 100 µl of anti‐DIG‐POD (final unit: 60 mU/ml, Roche Diagnostics) antibodies suspended in the wash buffer were added to each well and the plate was incubated for 1 hr at 37°C. After washing three times again, a Clarity Western ECL^®^ substrate (Bio‐Rad) (100 µl) was added and the plate was incubated for 1 min at R.T. The luminescence was measured by a plate reader (Promega GloMax, GM3000). The assay was performed at least three times, and the data are shown as the mean value with the standard deviation.

### 4.4 Epsilon RNA WT‐dependency assay

For the wild‐type ε RNA‐dependency experiment, the 5 pmol ε RNA (WT)‐DIG per 100 µl reaction buffer was serially diluted by 10‐fold as indicated in Figure 2d. Then, 5 pmol ε mutant RNA (ΔBΔL) was also reacted with the Strep‐TP‐spacer‐RT‐His8 protein in a similar fashion and the samples were incubated at R.T. for 30 min. After washing 3 times, 100 µl of anti‐DIG‐POD (final concentration: 60 mU/mL) antibodies were added and the plate was incubated at 37ºC for 1 hr. The luminescence was measured as described above. The assay was performed at least three times, and the data are shown as the mean value with the standard deviation.

### 4.5 Epsilon RNA‐binding competition experiment

For competition experiments, Strep‐TP‐spacer‐RT‐His8 proteins were fixed on streptavidin‐coated plates as above, and the binding buffer including different concentrations of either nonlabeled ε RNA (WT) or ε ΔBΔL (10, 50 or 500 pmol/100 µl/well) was added with 5 pmol/well DIG‐labeled ε WT. After incubation at R.T. for 30 min, each well was washed with 250 µl of the washing buffer three times. Then, 100 µl of anti‐DIG‐POD (final concentration: 60 mU/ml) was added and the plate was incubated at 37°C for 1 hr. The luminescence was measured as described above. The assay was performed at least three times, and the data are shown as the mean value with the standard deviation.

### 4.6 Compound screening with the ε RNA‐binding assay

A small‐molecule compound library (LOPAC^®1280^; Sigma‐Aldrich LO4200) was used for screening. All the compounds were used at 100 µM for primary screening. Compounds were mixed with 5 pmol DIG‐labeled ε WT for the reaction in each well and incubated at R.T. for 3 hr. After washing three times with the wash buffer, 100 µl of anti‐DIG‐POD antibodies (final concentration: 60 mU/ml) were added and the wells were incubated at 37°C for 1 hr. The luminescence was finally measured as above. All the compounds which showed over 80% inhibition of binding between Strep‐TP‐spacer‐RT‐His8 and ε RNA were used for the second time screening. For the second time screening, each of the compounds was diluted threefold at the indicated concentration (see Figure 3). 6‐hydroxy‐DL‐DOPA (DL‐DOPA, Sigma H2380) and N‐oleoyldopamine (OLDA, Sigma O2139) were purchased from Sigma‐Aldrich. The assay was performed at least three times, and the data are shown as the mean value with the standard deviation.

### 4.7 Cells for the cell‐based analysis for compounds

A stable HBV‐producing cell line, HB611 cells (Tsurimoto et al., 1987) were cultured in DMEM (low glucose) (Nacalai Tesque) containing 10% heat‐inactivated fetal bovine serum (FBS) (Sigma‐Aldrich), 1% antibiotic‐antimycotic solution (Nacalai Tesque) and 0.5 mg/ml G418 disulfate (Nacalai Tesque).

Human NTCP expressing HepG2 cells (NTCP/G2) (Iwamoto et al., 2014) were maintained in a primary hepatocyte maintain media (PMM) (Williams’ E medium [Gibco] supplemented with 10% heat‐inactivated FBS, 1% antibiotic‐antimycotic solution [Nacalai Tesque], 50 µM hydrocortisone [Sigma‐Aldrich], 5 µM dexamethasone, 5 µg/ml transferrin [Wako], 10 ng/ml EGF [Thermo Fisher], 5 µg/ml insulin [Sigma‐Aldrich], 5 ng/ml sodium selenite, 2 mM L‐glutamine [Nacalai Tesque] and 0.5 mg/ml G418 [Nacalai Tesque]).

HepAD38.7 cells (Ladner et al., 1997) were maintained in a DMEM/F12 (Nacalai Tesque) supplemented with 10% heat‐inactivated FBS, 1% antibiotic‐antimycotic solution (Nacalai Tesque), 5 µg/ml insulin, 0.5 mg/ml G418 and 400 ng/ml tetracycline (tet). To obtain HBV for HBV infection experiment, HepAD38.7 cells were induced for HBV replication by omitting tetracycline in the culture medium. The culture medium of confluent HepAD38 cells without tet was accumulated every week for 2 weeks, and HBV was precipitated with 6% PEG8000 (final concentration) at 4°C overnight. The precipitates were pelleted by centrifugation, and the precipitated HBV was dissolved in PBS and concentrated followed by filtration through 0.45‐µm filter (Millipore). The HBV DNA was quantified by qPCR, and HBV at 500 GEI was used for infection experiment (Ladner et al., 1997).

### 4.8 Cell viability assay

HB611 cells were seeded at 500 cells/well (a collagen‐coated 96‐well clear bottom white plate, Corning) and NTCP/G2 cells were seeded at 3,000 cells/well in the same 96‐well plate in the 100 µl respective culture medium and were incubated with compounds at the indicated concentration (see Figure 4 or Figure 5a,b) for 6 days or 9 days, respectively. NTCP/G2 cells were cultured in the 2% dimethyl sulfoxide (DMSO) containing PMM. CellTiter‐Glo^®^ luminescent cell viability assay solution (Promega) (100 µl) was mixed in the culture medium and incubated at 37°C for 30 min. Luminescence was measured with a plate reader (Promega, GloMax^®^, GM3000) according to the manufacturer's direction. The 50% cytotoxic concentration (CC_50_) was defined as the compound's concentration (µM) required for the reduction of cell viability by 50%, which was calculated by regression analysis (see figure legends of Figure 4).

### 4.9 Cell‐based analysis for compounds

HB611 cells were seeded at 2000 cells/well (a collagen‐coated 24‐well plate, Iwaki Techno Glass) in the 500 µl culture medium and treated with either 6‐hydroxy‐DL‐DOPA (DL‐DOPA) (6.25, 12.5 and 25 μM) or N‐oleoyldopamine (OLDA) (39, 78 and 156 nM) or 20 nM entecavir (ETV). Drug‐containing culture medium was refreshed every 3 days for 6 days. The culture supernatants and the cells at the 6th day were collected, HBsAg and HBeAg in the supernatant were measured by ELISA (see below), and the intracellular core‐associated and the extracellular particle‐associated HBV DNA were prepared for qPCR (see below).

In case of NTCP/G2, the cells were seeded at 2 × 10^5^ cells/well (a collagen‐coated 24‐well plate, Iwaki Techno Glass) one day before infection and infected with HBV obtained from HepAD38.7 at about 500 genome equivalent of infection (GEI) in 4% PEG 8000–2% DMSO containing PMM (500 µl) in the presence or absence of compounds. The infected cells were washed 2 times with PMM containing 2% DMSO 24 hr after infection, and either 6‐hydroxy‐DL‐DOPA (DL‐DOPA) (6.25, 12.5 and 25 μM) or N‐oleoyldopamine (OLDA) (3.125, 6.25 and 12.5 μM) or 20 nM entecavir (ETV) was added to the medium. Drug‐containing culture medium was replaced every 3 days for 9 days, the culture supernatants and the cells on the last day were collected, HBsAg and HBeAg in the supernatants were measured by ELISA, and the intracellular core‐associated and the extracellular particle‐associated HBV DNA were prepared for qPCR.

### 4.10 DNA extraction from intracellular core and extracellular particles

The supernatant from drug‐treated cells was incubated with 20 U/ml DNase I (Takara) in the presence of 5 mM MgCl_2_ for 2 hr at 37°C to degrade DNA outside the particles. The reaction was stopped by adding 10 mM EDTA pH 8.0 as the final concentration. Virus particles were precipitated with 6% (w/v) PEG 8000 (Sigma‐Aldrich). The precipitate was suspended in a buffer containing 10 mM Tris‐HCl (pH 7.6), 5 mM EDTA (pH 8.0) and 0.5% SDS and 0.2 mg/ml proteinase K (Roche) and incubated for 3 hr at 56°C, and then RNase A (Roche) was added at 0.4 mg/ml and incubated for further 30 min at 65°C. The virion‐associated HBV DNA was extracted by PCI (phenol: chloroform: isoamyl alcohol = 25:24:1) with 10 µg sonicated salmon sperm DNA and precipitated with ethanol in the presence of 0.25 mg/ml glycogen (Nacalai Tesque) as a carrier. The pellet was finally suspended in 20 µl TE (10 mM Tris‐HCl [pH8.0], 1 mM EDTA [pH8.0]).

For core‐associated HBV DNA preparation, cells were harvested and washed with PBS and pelleted at 7,200× *g* 5 min at 4°C. The cell pellet was vigorously suspended in a hypotonic buffer (20 mM Tris‐HCl [pH 7.8], 50 mM NaCl, 5 mM MgCl_2_ and 0.1% 2‐mercaptoethanol). The cell debris was excluded by centrifugation at 7,200× *g* for 5 min at 4°C. This cleared lysate was treated as the virion‐associated HBV DNA preparation, and the core‐associated HBV DNA was finally prepared in 20 µl TE as above.

### 4.11 Extraction of core‐/particle‐associated RNA

To evaluate packaged RNA, cleared lysates of drug‐treated cells were prepared as the core‐associated HBV DNA preparation at the end of the assay in both cases of NTCP/G2 and HB611 cells. Then, the lysate was added three volumes of TRIzol™ (Invitrogen) and RNA was extracted according to the manufacturer's direction except that 1 µg of yeast tRNA was added just before PCI extraction and 0.25 mg/ml glycogen solution (Nacalai Tesque) was added just before isopropanol precipitation. The RNA was finally suspended in 20 µl TE and stored as core‐associated HBV RNA at −70°C freezer until use.

### 4.12 Quantitative real‐time PCR (qPCR) and reverse transcription followed by qPCR (RT‐qPCR)

For the core‐associated HBV DNA and the extracellular particle‐associated HBV DNA, 1 µl from the core‐associated HBV DNA and the extracellular particle‐associated HBV DNA preparation (20 µl in total) was subjected to qPCR using Fast SYBR^TM^ Green Master Mix (Applied Biosystems‐Thermo Fisher Scientific), and a following qPCR primers were set at the S region (forward; 5'‐ CTTCATCCTGCTGCTATGCCT −3', reverse; 5'‐ AAAGCCCAGGATGATGGGAT −3'). The qPCR was monitored with QuantStudio™ 6 Flex (Applied Biosystems by Life Technologies). The PCR condition was 95°C for 20 s initially, then 40 cycles of 95°C for 1 s and 60°C for 20 s, and finally 1 cycle of 95°C for 15 s, 60°C for 1 min, 95°C for 30 s and 60˚C for 15 s.

For reverse transcription reaction, 10 µl of core‐associated HBV RNA was mixed with 10 ng random primers and 0.5 mM dNTPs and quickly chilled on ice after heat denaturation at 65˚C for 5 min. The solution was arranged in a 20 µl reaction mixture containing 5 mM DTT, 40 units of RNasin^®^ (Promega) and 200 units of superscript III (Invitrogen) and then incubated at 37°C for 30 min, 42 min for 30 min and 50°C for 60 min. Finally, the mixture was incubated at 70°C for 30 min to inactivate reverse transcriptase. One microliter of the reversely transcribed sample was subjected to qPCR for which primers were set at the HBV precore–core region (forward; 5'‐ TAATCATCTCATGTTCATGTCCTA‐ 3' and reverse; 5'‐ GAGATCTCGAATAGAAGGAAAG −3') with the same condition as above.

Copy number was determined according to the standard copies using a plasmid containing one HBV genome. The qPCR or RT‐qPCR experiment was performed at least three times, and the data are shown as the mean value with the standard deviation relative to the control.

### 4.13 Enzyme‐linked immunosorbent assay (ELISA)

HBsAg and HBeAg in culture supernatants were analyzed by using the HBs Antigen Quantitative ELISA Kit Rapid‐II (Beacle Inc.) and the e Antigen ELISA Kit (Bioneovan Co.), respectively.

### 4.14 Antibodies

An anti‐His‐tag antibody (Nacalai Tesque) and an anti‐SUMO antibody (Life Sensors) were used for protein detection by Western blotting. An anti‐digoxigenin (DIG)‐POD (Roche) antibody was used for digoxigenin‐labeled ε RNA detection in the in vitro assay.

### 4.15 Statistical analysis

Statistical significance was assessed using analysis of variance (ANOVA) with a threshold of *p < .05, **p < .01, ***p < .005 or ****p < .001. Values of *p* > .05 were regarded as nonsignificant (N.S.).

## CONFLICT OF INTERESTS

The authors declare that the research was conducted in the absence of any commercial or financial relationship that could be construed as a potential conflict of interest.

## Supporting information

Supinfo
