# Supplementary material for: Establishment of a system for finding inhibitors of ε RNA binding with the HBV polymerase
Source: Genes Cells. 2020 Jun 8;25(8):523–37. doi: 10.1111/gtc.12778 (PMC7496097; doi:10.1111/gtc.12778)

SUPPLEMENTARY DATA

Supplemental Methods

Protein expression and purification

The HBV polymerase terminal protein (TP) domain, which contains amino acids 1 to 177 was cloned into a pE-SUMO vector (nacalai tesque) that was designed to express SUMO-TP protein tagged with 6x His (His6-SUMO-TP) at the N-terminus. The parental vector was used to express the His6-tagged SUMO protein.

E. coli cells of the strain Rosetta gami2 (DE3) were transformed by these vectors. The transformed cells were grown at 37°C until the culture reached OD_{600} 0.3 and induced for protein expression with 1 mM isopropyl- β-D-thiogalactopyranoside (IPTG) for 6 hours. The *E. coli* pellets were suspended in a lysis buffer (100 mM Tris-HCl [pH 8.0], 0.1% Triton X-100, 300 mM NaCl, 0.1% protease inhibitor cocktail and 1% SDS), and sonicated for 20 seconds five times with 30 second intervals on ice. The cell lysate was incubated at 4ºC for overnight, and then centrifuged at 12,000 × g for 40 min to remove the SDS. The supernatant was then passed through with a 0.45 μm filter (Millipore), and incubated with IMAC® Ni-charged resin (Bio-Rad) to purify the strep-TP-spacer-RT-8His by affinity to the resin. The protein-bound resin was washed with a washing buffer (100 mM Tris-HCl [pH 8.0], 0.1% sarkosyl, 300 mM NaCl, 5 mM imidazole, and 0.1% protease inhibitor cocktail [Sigma-Aldrich]) five times and then eluted with an elution buffer (100 mM Tris-HCl, [pH 8.0], 0.1% sarkosyl, 300 mM NaCl, 150 mM imidazole, and 0.1% protease inhibitor cocktail).

The purified proteins were mixed with 2 mg/mL NV10® and incubated for overnight at room temperature (R.T.) for refolding in a buffer containing 100 mM Tris-HCl (pH 8.0), 300 mM NaCl, 1 mM DTT and 0.1% protease inhibitor cocktail (Sigma-Aldrich) using a slide-A-lyzer dialysis cassette system (10kDa) (Thermo). His6-SUMO protein was expressed in the same way. The cell pellet was suspended in another lysis buffer (50 mM NaH_2PO_4 [pH 8.0], 0.1% Triton X-100®, 300 mM NaCl, 1 mM 2-mercaptoethanol [Wako], 1 mg/mL lysozyme, 125U of benzonase [Novagen] and 0.1% protease inhibitor), and was sonicated for 20 seconds 5 times with 30 second intervals on ice. The cell lysate was centrifuged at 12000 × g for 40 min at 4˚C. The supernatant was passed through a 0.45 µm filter (Millipore), and then the HIS6-SUMO protein was purified using the Profinity IMAC® Ni-charged resin. The resin bound with His6-SUMO was washed with a washing buffer (50 mM NaH_2PO_4 [pH 8.0], 0.1% Triton X-100®, 300 mM NaCl, 10% glycerol, 5 mM imidazole, 0.1% protease inhibitor) five times and then bound His6-SUMO was eluted with an elution buffer (50 mM NaH_2PO_4 [pH 8.0], 0.1% Triton X-100®, 300 mM NaCl, 10% glycerol, 150 mM imidazole, protease inhibitor).

***In vitro* ε RNA-binding assay**

For the ε RNA binding assay using His6-SUMO and His6-SUMO-TP proteins, 50 pmol of the purified proteins was fixed on a nickel-coated 96-well plate (Pierce) and were incubated at R.T. overnight. Then, 100 µl of 1 mg/mL BSA in phosphate buffered saline (PBS) was added and the plate was incubated for 1 hour at 37˚C to

block non-specific binding. Next, 5 pmol of DIG-labelled either ε WT or ε ΔBΔL was added to the protein-coated plates in a 100 μl reaction buffer (50 mM Tris-HCl [pH 7.5], 150 mM NaCl, 1 mM EDTA, 0.05% NP 40, 0.1% protease inhibitor cocktail and 2 mM DTT). After 3 hours incubation at R.T., each well was washed with 250 μl of wash buffer (100 mM Tris-HCl [pH 8.0], 300 mM NaCl, 1 mM $MgCl_2$, 5 mM 2-mercaptoethanol and 50 μg/mL BSA) 3 times and then 100 μl of anti-DIG-POD (final concentration: 60 mU/mL) in the wash buffer was added and the well was incubated for 1 hour at 37˚C. After washing 3 times again, a Clarity Western ECL® substrate (100 μl) was added and the well was incubated for 1 min at R.T. Finally, the luminescence was measured by a plate reader (Promega GloMax, GM3000).

Compound screening with the ε RNA binding assay

A small molecule compound library (LOPAC®1280; Sigma-Aldrich LO4200) was used for screening. All the compounds were used at a concentration of 100 μM for the primary screening; for the reaction, all compounds were mixed with ε RNA and incubated at R.T. for 3 hours. After washing 3 times, 100 μl of anti-DIG-POD (final concentration: 60 mU/mL) was added and incubated at 37˚C for 1hour. A Clarity Western ECL® substrate (BIO-RAD) (100 μl) was added and incubated for 1 min at R.T. Finally, the luminescence was measured by a plate reader (Promega GloMax, GM3000).

HB611 cell and NTCP/G2 cell morphology in the presence of drugs

The HB611 cell and NTCP/G2 cell (infected at 500 GEI) were treated with drugs as described (see the Cell-based analysis for compounds in the Materials and Methods). After 6 days for HB611 and 9 days for NTCP/G2 cells, the cell morphology was observed with a microscopy (EVOS FL Auto microscope, Life technologies).

Affinity determination by surface plasmon resonance (SPR)

The *K*D values were determined with a BIAcore® T200 (GE Healthcare) at 25°C in a running buffer (PBS - 0.05% Tween 20) in the presence of 20 μg/ml refolded Strep-TP-spacer-RT-His8 or Strep-GFP-His8 was immobilized onto a CM5 sensor chip (GE Healthcare), and the interaction of the proteins with compounds; DL-DOPA, OLDA, ETV and Dopamine was detected by monitoring injections of 200, 100, 50, 25, 12.5, 6.25, 3.125, 1.56, 0.78 and 0.39 μM (diluted with the running buffer) in the kinetic injection mode. Measurement conditions were 30 μl/min flow rate, 120 s for the compound injection time and 200 s for the dissociation monitoring time. After each injection, the sensor surface was regenerated twice with a 30 μl injection of 2M NaCl - 5% Triton- X100®. To determine the *K*D values, sensorgrams of both a reference cell (no protein immobilization) and a measurement with the running buffer injection were subtracted from each sensorgram of the protein. The data were fitted with a 1: 1 binding model, using the BIA evaluation T200 software (GE Healthcare).

Antibodies. An anti-SUMO chicken-IgY (Life Sensors, Cat. # AB7002) and a Goat anti-Chk IgY conjugated with HRP (abcam, Cat. # ab97135) were used for protein detection by Western blotting and *in vitro* assay.

Supplemental Figure legend

Supplementary Figure 1. Purification of recombinant HIS6-SUMO-TP, and HIS6-SUMO control protein. **(A and B)** Left: CBB stained HIS6-SUMO-TP and HIS6-SUMO control protein. Finally, HIS6-SUMO-TP and HIS6-SUMO protein with the purity over 90% were successfully obtained. Right: Western blots of HIS6-SUMO-TP-spacer and HIS6-SUMO control protein. For Western blotting, a chicken anti-SUMO IgY against the SUMO tag was used to detect each protein. **(C)** ε RNA binding assay. Left: The principle of binding evaluation of HIS6-SUMO and HIS6-SUMO-TP (un-refold or refolded). The proteins were bound to the Nickel coated plate and the relative binding activity between HIS6-SUMO-TP-spacer and HIS6-SUMO (un-refold or refolded) protein binding to the streptavidin plate (right panel). **(D)** ε RNA binding assay. Left: The principle of HIS6-SUMO-TP and HIS6-SUMO (un-refold or refolded) protein binding assay. After fixing proteins on the plate, 5 pmol ε WT was added. The relative binding activity between HIS6-SUMO-TP-spacer or HIS6-SUMO (un-refold or refolded) protein binding to the wild type (WT) ε RNA or the mutant (MT) ε RNA (right panel). All the sample were treated with Clarity Western ECL Substrate (BIO-RAD). The luminescence was measured by a plate reader (Promega GloMax, GM3000). Data we from one representative of at least three independent experiments; the means and S.D. of triplicate experiment are show (N. S.: not significant)

Supplemental Figure 2. (**A**) The chemical compounds screening. The small molecule compound library (LOPAC$^{®1280}$; Sigma-Aldrich LO4200) was used for the screening. All the compounds were made 100 μM mixed with ε RNA-WT-Dig for reaction with Strep-TP-RT-HIS8 protein. The reaction methods and materials all the same as described in previously, the detail see the *In vitro* RNA binding assay. (**B**) The chemical structure of DL-DOPA, OLDA and Dopamine

Supplemental Figure 3. (A) The HB611 cell morphology. After 3 days treated the different concentrations of drug or control DMSO, Entecavir (ETV), the cell morphology was checked by a microscope with 10-time and 20-time magnification. **(B)** The NTCP/G2 cell after infected by HBV virus 500 GEI, then treated the different concentrations of drug or control DMSO, Entecavir (ETV), the cell morphology was checked as same as HB611 cell.

Supplemental Figure 4. Affinity analysis of Strep-TP-spacer-RT-HIS8 and Strep-GFP-HIS8 with compounds by SPR. The binding affinity of the refolded Strep-TP-spacer-RT-HIS8 or Strep-GFP-HIS8 proteins and four compounds was determined by BIACORE® T200 at 25°C in the running buffer (PBS - 0.05% Tween 20). Strep-TP-spacer-RT-HIS8 **(A, B, C, D)** or Strep-GFP-HIS8 (**E** and **F**) was immobilized onto a CM5 sensor chip at the concentration of 20 μg/ml, and the compounds DL-DOPA, OLDA, ETV, and dopamine was injected at concentrations of 200, 100, 50, 25, 12.5, 6.25, 3.125, 1.56, 0.78, and 0.39 μM. Left panels: The refolded Strep-TP-spacer-RT-HIS8 protein binding to DL-DOPA, OLDA, and two negative control ETV and

dopamine by kinetic mode. Right panels: The results presented by affinity mode. All the graphs were automatically generated by BIA evaluation T200 software (GE Healthcare). In case of Strep-GFP-HIS8, two compounds; DL-DOPA and OLDA were analyzed.

Supplementary Fig. 1

A

His6-SUMO-TP-spacer

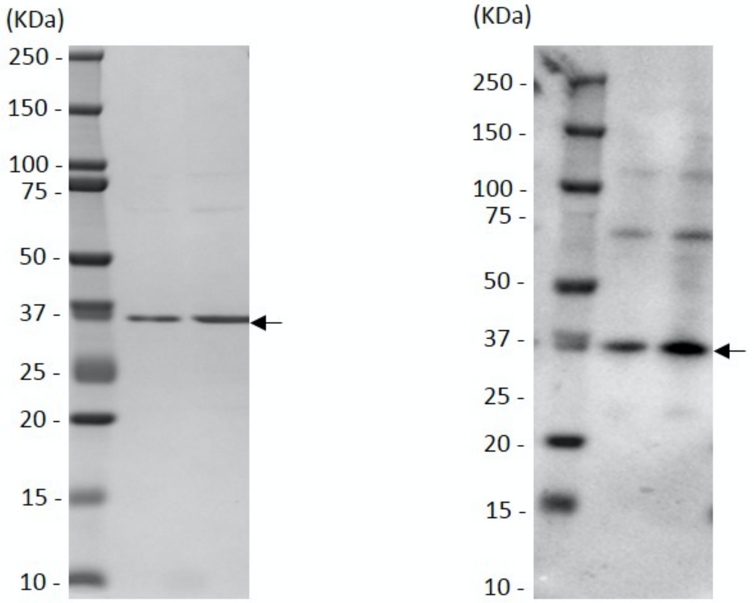

B

His6-SUMO

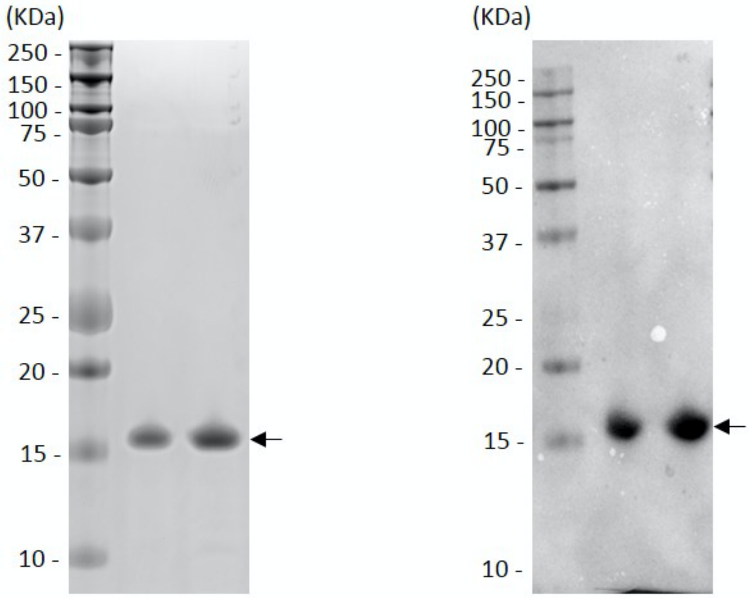

Supplemetary Fig. 1

C

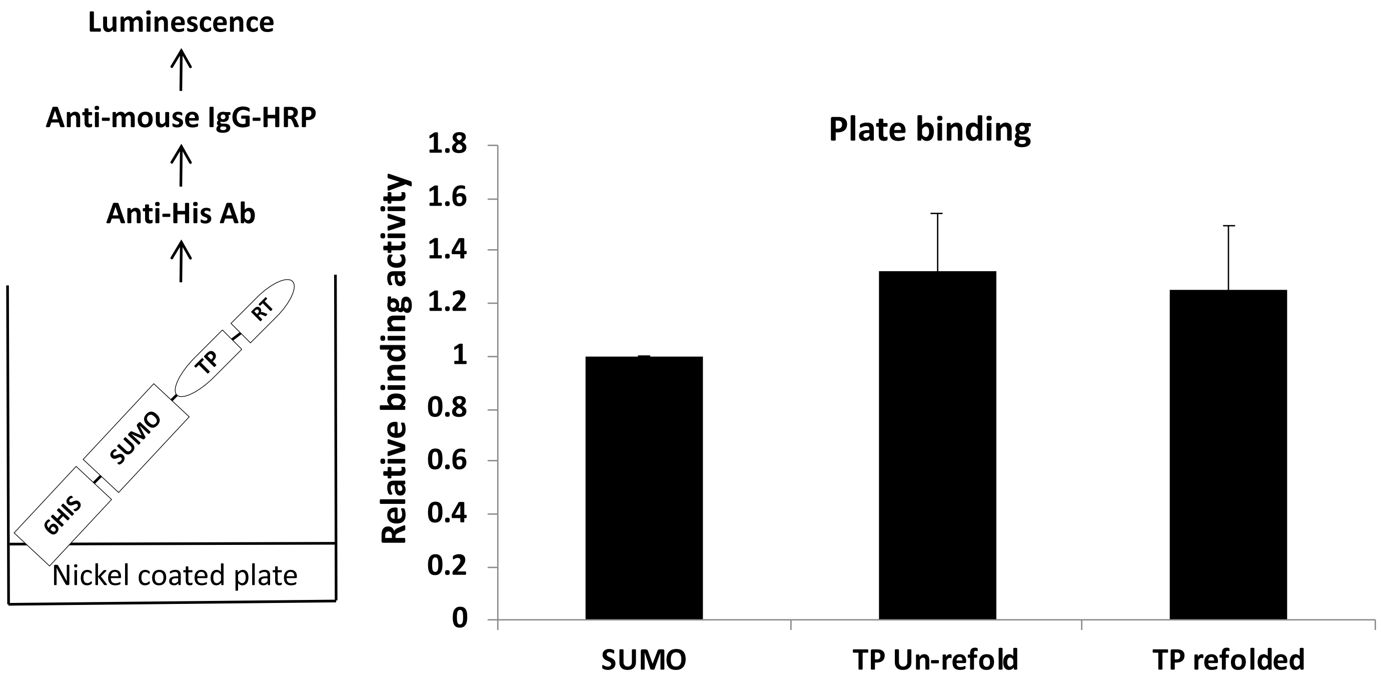

D

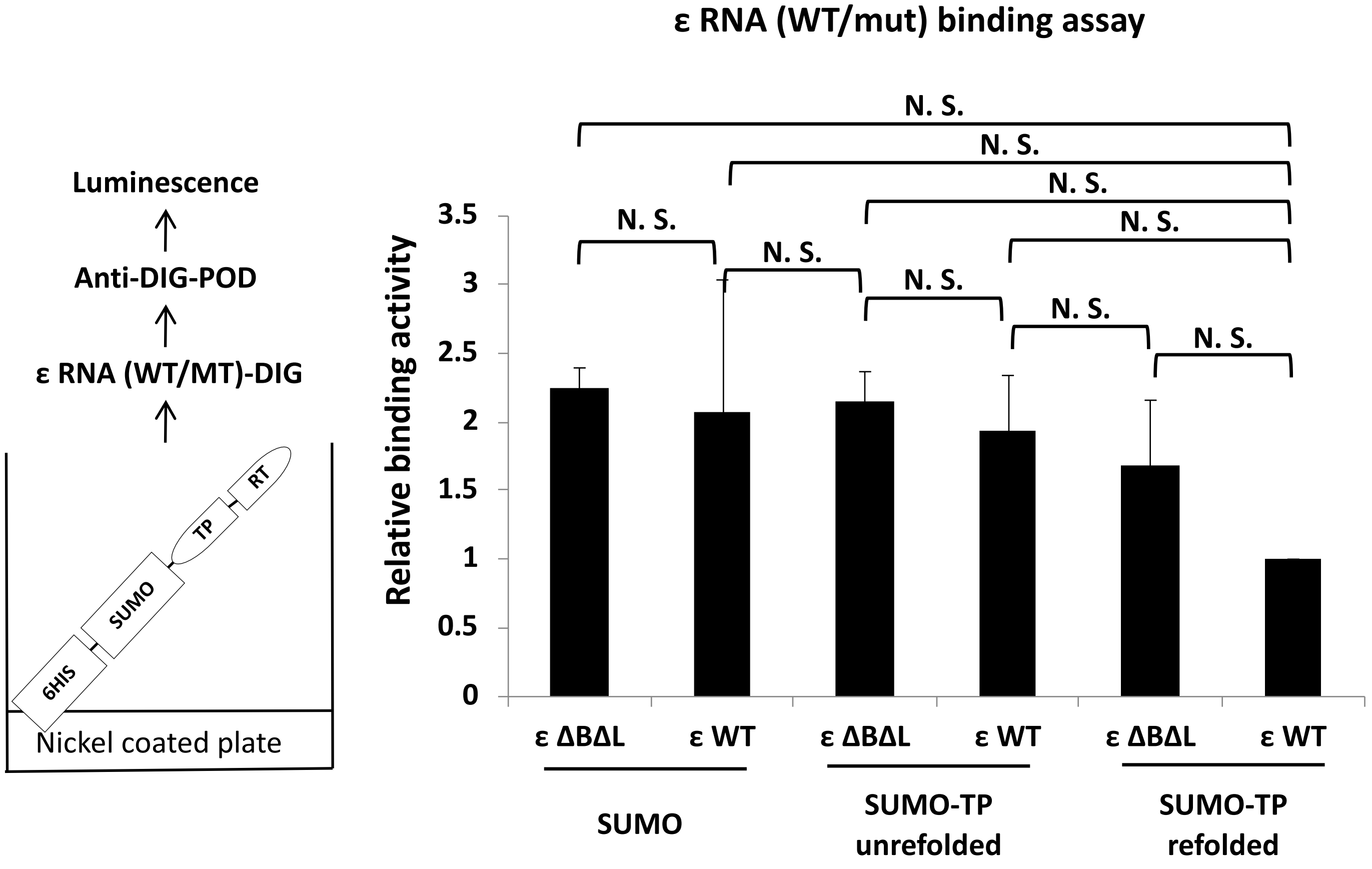

Supplementary Fig. 2

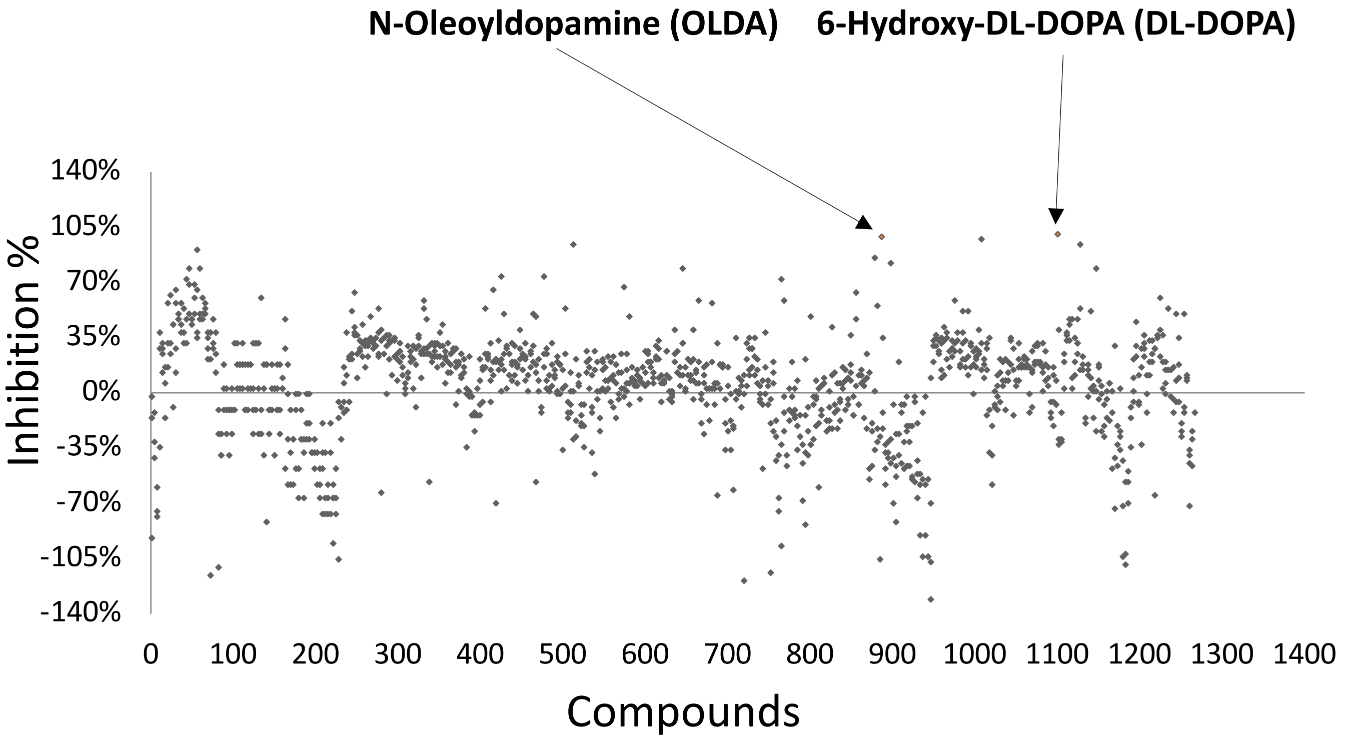

Supplementary Fig. 3.

A

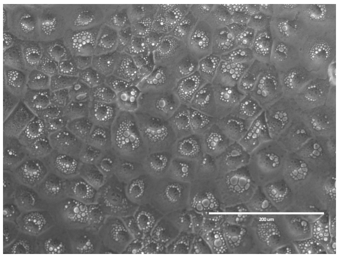

DMSO

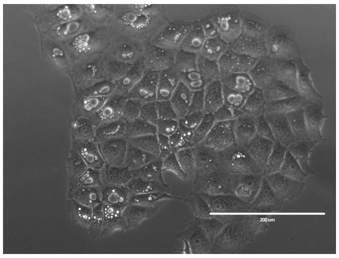

ETV

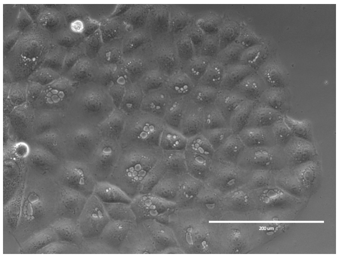

6.25 DL-DOPA

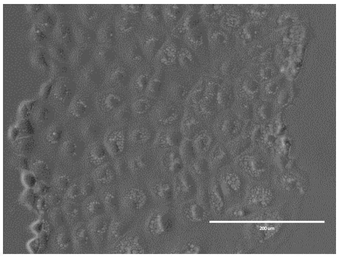

0.039 OLDA

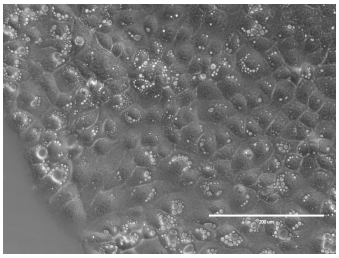

12.5 DL-DOPA

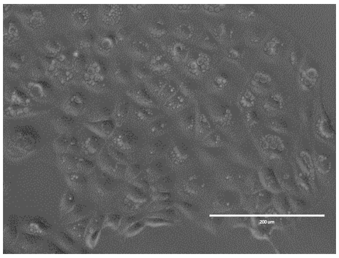

0.078 OLDA

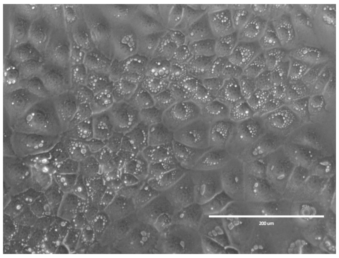

25 DL-DOPA

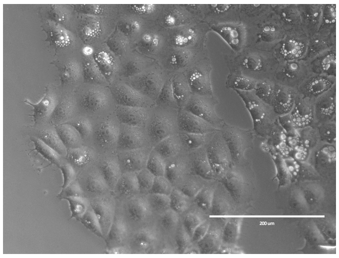

0.156 OLDA

Supplementary Fig. 3.

B

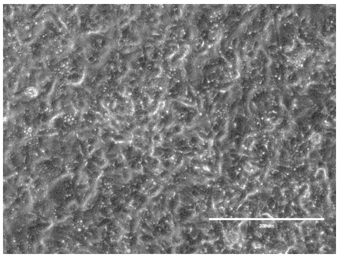

DMSO

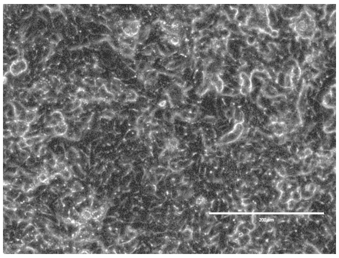

20 nM ETV

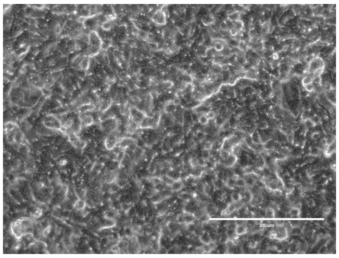

6.25 µM DL-DOPA

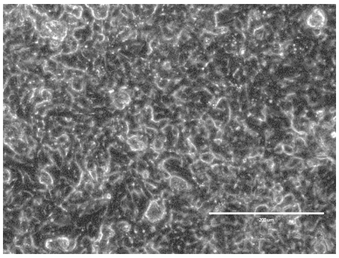

3.125 µM OLDA

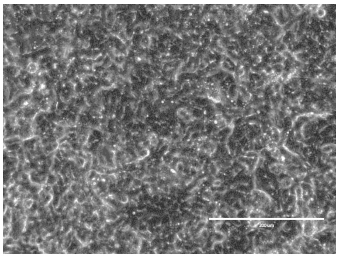

12.5 µM DL-DOPA

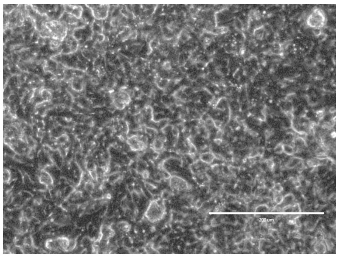

6.25 µM OLDA

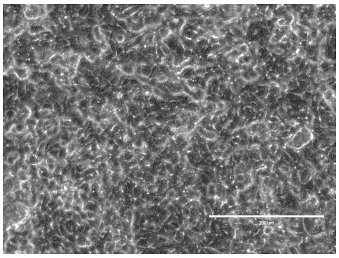

25 µM DL-DOPA

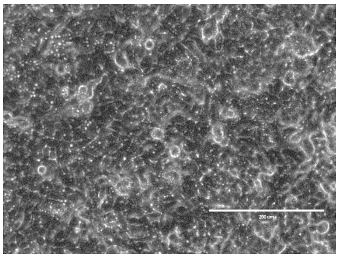

12.5 µM OLDA

Supplementary Fig. 4

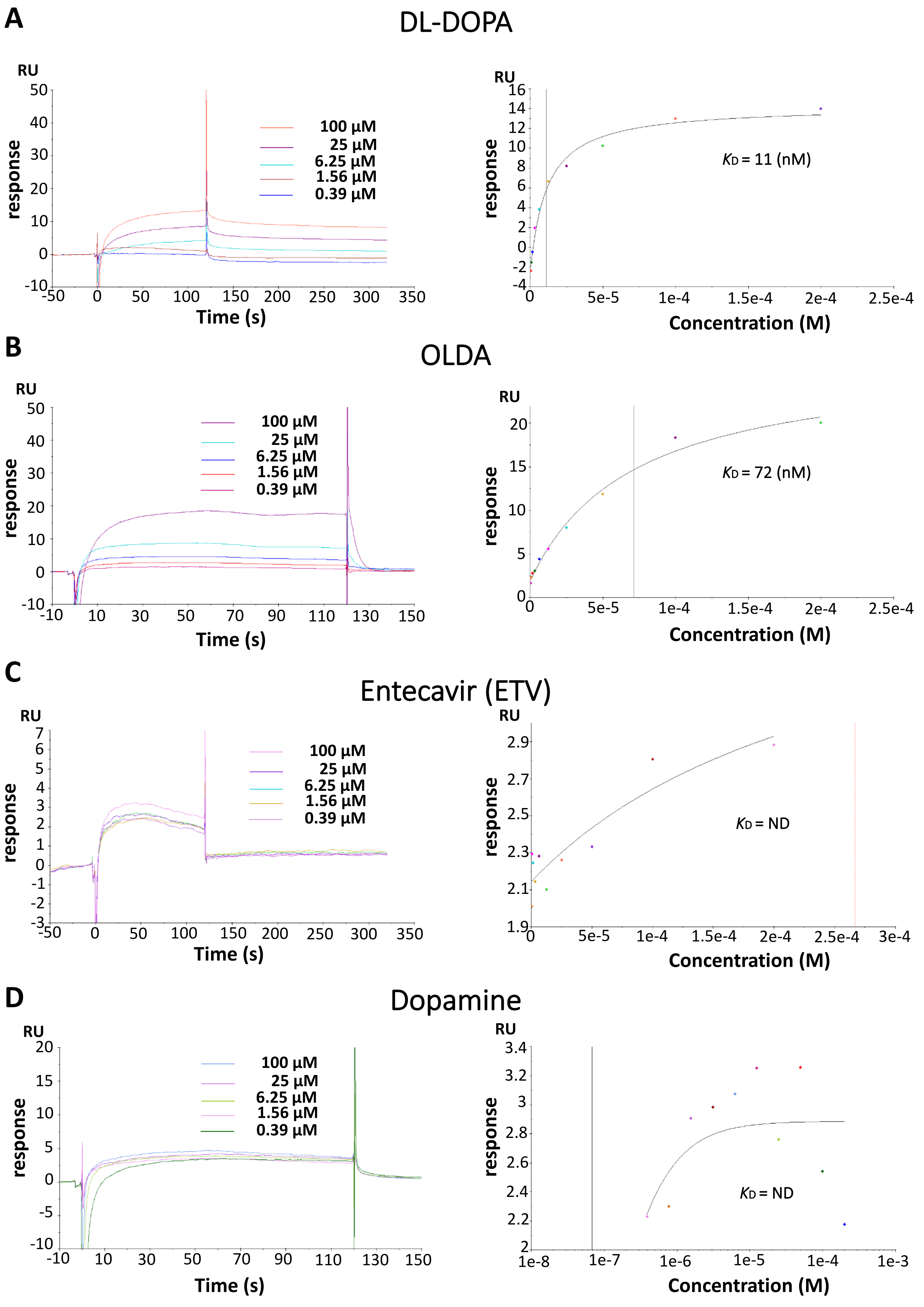

Supplementary Fig. 4

E

DL-DOPA

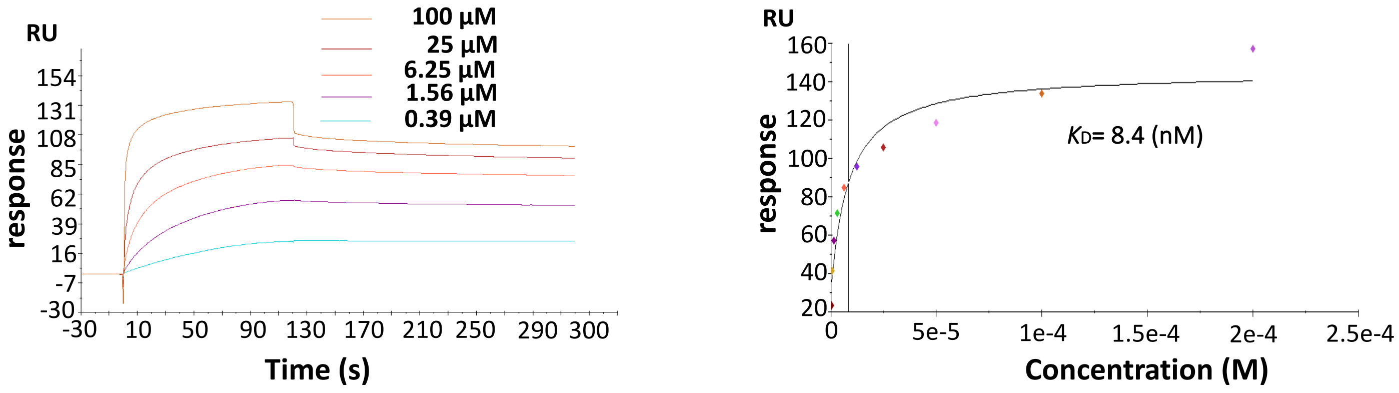

F

OLDA

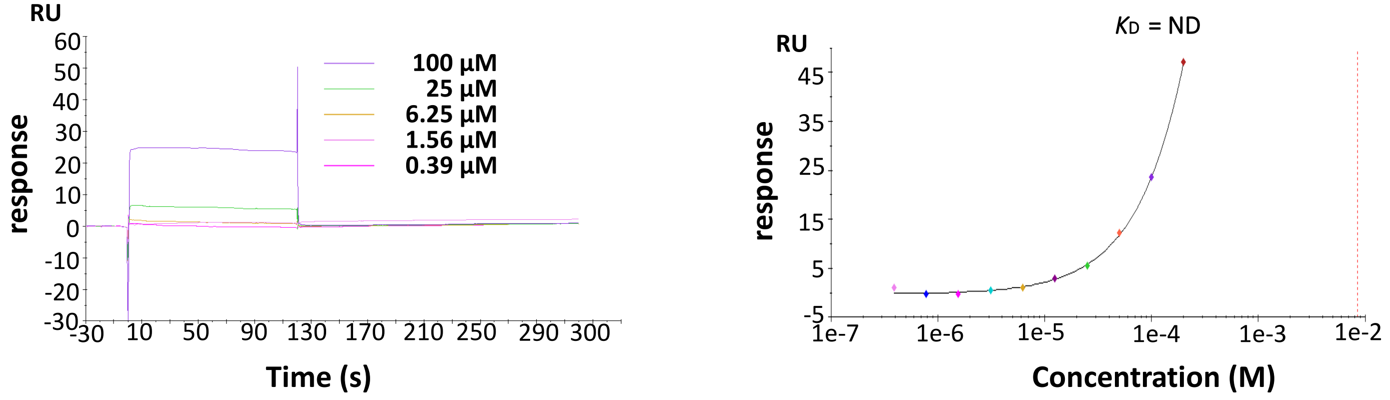

Supplement: Supplementary file 1 — Supinfo [file GTC-25-523-s001.pdf]
